# Construction Worker Risk-Taking Behavior Model with Individual and Organizational Factors

**DOI:** 10.3390/ijerph16081335

**Published:** 2019-04-13

**Authors:** Banus Kam Leung Low, Siu Shing Man, Alan Hoi Shou Chan, Saad Alabdulkarim

**Affiliations:** 1 Department of Systems Engineering and Engineering Management, City University of Hong Kong, Hong Kong, China; kllow2-c@my.cityu.edu.hk (B.K.L.L.); alan.chan@cityu.edu.hk (A.H.S.C.); 2 Industrial Engineering Department, College of Engineering, King Saud University, Riyadh, P.O. Box 145111, Saudi Arabia; Alabdulkarims@ksu.edu.sa

**Keywords:** construction safety, construction workers, individual factors, organizational factors, risk-taking behavior

## Abstract

Behavioral-based safety is an important application of behavioral science that can be used to address safety problems in the construction sector. An understanding of construction worker risk-taking behavior is deemed to be a crucial basis on which concerned authorities and construction companies can develop effective safety interventions to reduce construction accidents. However, no studies have been conducted to examine the effects of safety climate, work condition, attitude toward risk, cognitive bias, and risk perception on construction worker risk-taking behavior through a quantitative approach. Accordingly, this study aims to propose a research model that explains construction worker risk-taking behavior. A total of 188 valid datasets were obtained through a series of questionnaire surveys conducted in representative construction projects in Hong Kong. Confirmatory factor analysis with structural equation modeling was adopted to validate the hypothesized research model. Results show that attitudes toward risk and cognitive bias have a positive influence, whereas risk perception and work conditions have a negative influence on construction worker risk-taking behavior. In addition, safety climate was negatively correlated with construction worker risk-taking behavior. Practical recommendations for reducing construction worker risk-taking behavior are also discussed in this paper.

## 1. Introduction

Despite the considerable improvement of occupational safety in Hong Kong over the past decade, a vast number of construction accidents have been contributing to the high proportion of industrial accidents and injuries [1]. In 2017, construction accidents and fatalities in Hong Kong reached 3902 and 22, respectively [1]. Similar phenomena were also observed in other regions (e.g., the UK and US) [2]. These facts indicate the urgent need for an improvement in construction safety performance.

The factors that influence the occurrence of construction accidents have been widely investigated. For instance, Rowlinson and Jia [3] analyzed factors that contribute to heat illness incidents, and found that these factors (such as local weather, societal culture, industrial coordination, training, project leadership, team culture, financial incentives, and fatigue status) can be classified into eight levels of actors, including the ecosystem, society, industry, permanent organization, project organization, team, job unit, and individual levels. Tsang et al. [4] developed a forecasting model to examine potential accident-related factors in the construction industry and their relationship with accident rates. They identified three types of accident-related factors, such as working conditions, environmental factors, and management actions, which can explain 56.7% of construction accident rates. Mistikoglu et al. [5] used the decision tree methods to analyze fall accidents experienced by roofers, and found that fall distance was the most important attribute in predicting roofer fall accident results (i.e., a fatality or nonfatal injury).

Different approaches have been developed by safety researchers to improve construction safety. For example, virtual reality technology has been used in construction engineering education and training [6]. Liu et al. [7] identified critical success factors for safety management in subway construction. In addition to technology- and management-based approaches, the behavior of construction workers has been an important safety research topic in the relevant literature [8], because behavior-based approach can be an effective way to avoid construction accidents [9]. Risk-taking is a type of unsafe behavior that involves making a decision with uncertainty in both the probability of failure/success and its associated severity [10]. Tixier et al. [11] investigated the psychological antecedents of risk-taking behavior of construction workers, and found that workers who were unhappy, sad, anxious, fearful, and disgusted were less likely to take risks at work than those who were amused, happy, interested, and joyful, because they had a higher level of risk perception. Bohm and Harris [12] found that risk-taking behavior of construction site dumper drivers was often influenced by situational factors, such as coworker behavior or site safety rules, together with a poor safety culture that gave priority to production. Man et al. [13] have recently accentuated the importance of understanding construction worker risk-taking behavior in improving construction safety performance.

Although previous studies on risk-taking behavior of construction workers have mainly adopted a qualitative approach, no quantitative studies have been conducted to examine the effect of factors that may influence construction worker risk-taking behavior. Thus, this study aims to fill this research area by proposing a research model accounting for construction worker risk-taking behavior. In this model, two domains of factors were involved—namely, individual and organizational factors, because they have been widely considered in explaining construction worker behaviors [14,15,16]. The research objective of this study was to examine the influence of attitudes toward risk, cognitive bias, risk perception, safety climate, and work conditions on the risk-taking behavior of construction workers by using structural equation modeling. A literature review on individual and organizational factors, methodology, results, discussion, and conclusions is presented in the following sections.

## 2. Literature Review

**Figure 1:**
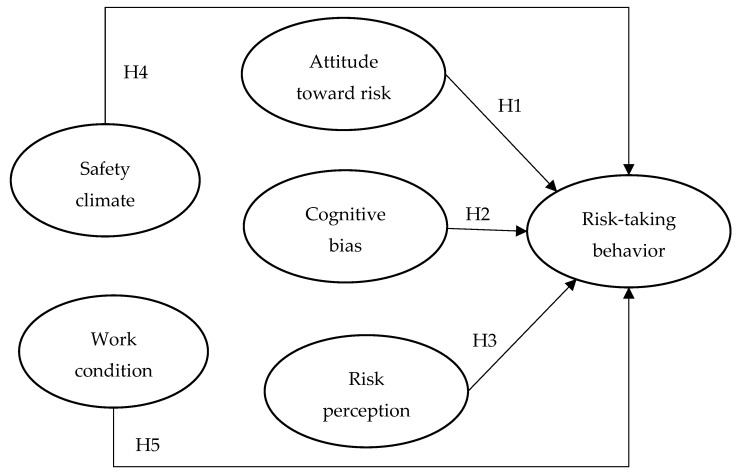
Proposed research model for explaining construction worker risk-taking behaviour to be tested in this study.

In the proposed research model (Figure 1), individual (including attitude toward risk, cognitive bias, and risk perception) and organizational factors (including safety climate and work conditions) explain construction worker risk-taking behavior. The construction of the proposed research model has a theoretical basis (i.e., the findings of previous relevant studies). The details of the development of hypotheses in the proposed research model are discussed in Section 2.1 and Section 2.2.

### 2.1. Individual Factors

Attitude is defined as the psychological tendency to evaluate a particular entity in terms of some degree of favor or disfavor [17]. Attitudes toward risk is defined as a person’s positive or negative evaluation of risks at work, and refers whether workers are risk-neutral, risk-aversive, or risk-seeking [18]. Attitudes toward risk is one of the essential elements in taking a risk [19]. This notion has also been widely applied to other areas of safety science, such as transportation safety [20]. Attitudes toward risk makes a significant contribution in predicting the risky behavior of drivers [20]. The mental shortcut of people in making judgments is commonly defined as cognitive bias, which includes overconfidence, illusion of control, and belief [21]. People with a high level of cognitive bias consistently tend to believe that they are less likely to experience a negative event themselves compared with others. Montibeller and Von Winterfeldt [22] suggested that cognitive biases can affect behavioral decisions, such as risk-taking behavior, which may violate commonly accepted normative principles. Risk perception is defined as the subjective judgment that one makes about the frequency and severity of particular risks [23]. It has been considered an important factor in explaining construction worker risk-taking behavior in qualitative studies [13,24]. However, no studies have been conducted to examine the effect of risk perception on construction worker risk-taking behavior through a quantitative approach. Moreover, construction workers who have a high level of cognitive bias and a low level of risk perception tend to take risks at work. Therefore, the following hypotheses related to the factors of attitude toward risk, cognitive bias, risk perception, and risk-taking behavior were developed in the proposed research model:

**H1:**  *Attitude toward risk has a positive influence on construction worker risk-taking behavior.*

**H2:**  *Cognitive bias has a positive influence on construction worker risk-taking behavior.*

**H3:**  *Risk perception has a negative influence on construction worker risk-taking behavior.*

### 2.2. Organizational Factors

In an organizational context, a three-level hierarchy consisting of managerial policy implementation (top level), procedural arrangement (middle level), and team culture with proper supervision (group level) was utilized to understand individuals’ safety performance [25]. This hierarchy helps determine the sub-factors associated with work accidents under occupational factors. Among the three levels, safety climate plays a crucial role in workers’ safety performance [26]. Safety climate is the reflection of the attitudes, beliefs, perceptions, and values that employees share in relation to safety [27]. In a previous qualitative study on the risk-taking propensity of construction workers [24], safety climate was found to be one of the main reasons why construction workers take risks at work. Apart from safety climate, work condition was found to be an important factor that influences construction worker risk-taking behavior [13]. Work condition includes workplace constraints and safety equipment availability. Poor working conditions have also been found to be associated with risk-taking tendencies and occupational injuries involving earthwork construction workers, such as coal miners [28]. Within good work conditions, where workplace constraints are minimized and safety equipment is always available for workers, workers tend not to take risks at work. Therefore, the following hypotheses related to the factors of safety climate, work condition, and risk-taking behavior were developed in the proposed research model:

**H4:**  *Safety climate has a negative influence on construction worker risk-taking behavior.*

**H5:**  *Work condition has a negative influence on construction worker risk-taking behavior.*

## 3. Methodology

### 3.1. Research Design

This study adopted a quantitative approach with a structured questionnaire to identify the significant constructs in the proposed research model. Through the development of sophisticated measurement tools, a confirmatory strategy was used to test the newly proposed theoretical model for explaining construction worker risk-taking behavior. The structured questionnaire was implemented through individual face-to-face interviews for the accuracy and quality of the results.

### 3.2. Questionnaire Design

A self-administered questionnaire was developed for the collection of empirical data. The questionnaire consisted of three parts. The first part measured individual and organizational factors, including attitude toward risk, cognitive bias, risk perception, safety climate, and work conditions. The second part measured risk-taking behavior. The third part collected demographic information, such as gender, age, education level, and marital status. The questionnaire was developed based on an extensive literature review on relevant topics, and was adapted from validated measurement scales. Some items were modified to fit the context of this study. Table 1 lists the items used in this study and its references. All items were measured with a seven-point Likert scale, ranging from strongly disagree (1) to strongly agree (7). It took about 10 minutes to complete the questionnaire. 

### 3.3. Participants

A total of 192 construction workers from 11 construction sites were randomly selected to participate in this survey, and 188 of them were valid (i.e., no missing values) and therefore suitable for further statistical data analysis. The valid response rate was over 97%. The duration of each survey was approximately 30 min to 45 min. To minimize potential response bias, before the commencement of the survey, the participants were informed that they had the right to quit the survey at any time and that the information collected would be handled with absolute anonymity and confidentiality. Accordingly, all participants provided their written consents.

Table 2 lists the demographic data of the participants, including their age, gender, education level, and marital status. The data showed that 83% were male, similar to the gender distribution of the targeted population [41]. 80.8% were aged 31 or above. In addition, 85.1% had attained at least lower secondary education, and 70.2% were married. 

### 3.4. Data Analysis

Structural equation modeling (SEM), a statistical methodology for analyzing empirical data that may have multiple variables, has been previously used in studies on construction worker safety behavior [14,15,42]. Specifically, SEM can estimate relationships between observed variables and their underlying constructs, as well as the relationships among constructs [43]. For instance, Seo et al. [15] used SEM to analyze the relationships between the individual and organizational factors that affect safety behaviors of temporary construction workers.

As suggested by Anderson and Gerbing [44], a two-step approach for conducting structural equation modeling (SEM) was adopted to analyze the collected data. The first step was to use confirmatory factor analysis (CFA) to examine psychometric properties of the scales, such as internal consistency reliability, convergent validity, and discriminant validity. Internal consistency reliability is the degree to which items in a scale measure the same construct [45]. Convergent validity is the correlation between two or more scores on scales, which are designed to measure the same or similar constructs [46] while discriminant validity refers to the extent to which the constructs differs from one another empirically [47].

The second step was to use structural equation modeling to examine the structural model for determining the strength and direction of the relationships among the theoretical constructs. In other words, structural equation modeling was used for the verification of the proposed model and for testing the developed hypotheses in the proposed research model. 

CFA was conducted using the Statistical Package for the Social Sciences 23 (IBM, Armonk, NY, USA) and Analysis of Moment Structures (AMOS) 23 (IBM, Armonk, NY, USA), while SEM was conducted using AMOS 23. 

## 4. Results

### 4.1. Measurement Model Assessment

Table 3 shows factor loading (FL), composite reliability (CR), average variance extracted (AVE), and Cronbach’s α values for all the constructs of the model. According to Farrell [48], FL can be generally referred to as the correlation between a construct and the item that is designed to measure the construct. AVE is the average amount of variation that is explained by a latent construct in the observed variables which are designed to measure the latent construct. AVE can be obtained by averaging squared FLs across all observed variables that which are designed to measure the latent construct. CR and Cronbach’s α are measures of the internal consistency of a scale [49]. To verify construct reliability, the values of Cronbach’s alpha and CR for constructs should exceed the recommended value of 0.7 [49]. Convergent validity was determined on the basis that the FL of items should exceed 0.7 and the AVE for each construct should be larger than 0.5 [50]. The results showed that the reliability and convergent validity of all constructs were acceptable. For assessing the discriminant validity of the constructs, the AVE value of a construct should exceed the variance shared between the construct and other constructs in the measurement model (i.e., the squared correlation between two constructs). Thus, the square root of the AVE should be larger than the inter-correlations in the corresponding columns and rows for demonstrating adequate discriminant validity [50]. Table 4 indicates that the square root of the AVE of constructs was larger than the corresponding inter-correlations, implying the acceptable discriminant validity of all the constructs. 

The measurement model fit was examined with four goodness-of-fit indexes, namely, the comparative fit index (CFI), Tucker–Lewis index (TLI), root mean square error of approximation (RMSEA), and standardized root mean residual (SRMR) [51]. CFI and TLI are comparative fit indices used to evaluate the fit of a model relative to a more restricted, nested baseline model [52]. RMSEA is one type of parsimony correction indices that incorporate a penalty for poor parsimony of a model, and is used to test how well the model, with unknown but optimally chosen parameter estimates, would fit the population covariance matrix [43]. SRMR is a kind of absolute fit index, and a measure of the difference between the correlations in the input matrix and the correlations predicted by the model [53]. The results also showed that CFI and TLI were 0.925 and 0.912, respectively, which met the requirement of 0.9 [50]. Moreover, RMSEA and SRMR were 0.046 and 0.063, respectively, which are smaller than the allowed maximum value of 0.07 [54]. These results suggested that the measurement model fit was satisfactory. In summary, the acceptable model fit, adequate reliability, and validity of the measurement model supported that the measurement model was appropriate for the subsequent analysis of the structural model.

### 4.2. Structural Model Assessment

**Figure 2:**
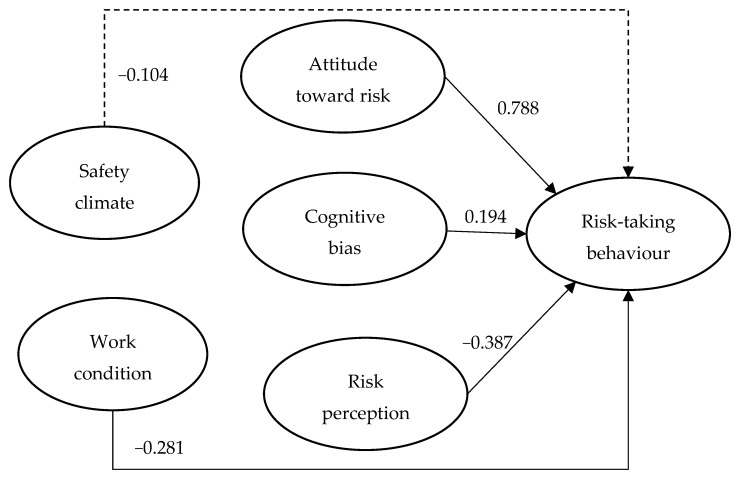
Results for the proposed research model in this study (solid line indicates significance and dotted line indicates non-significance).

To assess the structural model, the same four goodness-of-fit indexes (i.e., CFI, TLI, RMSEA, and SRMR) in the measurement model assessment were used. The values of CFI, TLI, RMSEA, and SRMR were 0.952, 0.928, 0.052, and 0.065, respectively, satisfying the cut-off criteria. The proposed research model can explain 65.5% of the variance in risk-taking behavior. The path analysis results of the hypotheses in the proposed research model are depicted in Figure 2. The path coefficients were estimated by a maximum likelihood algorithm that is available in AMOS 23, and which has been widely used in conducting SEM [43]. The *p*-values are different for the five hypotheses because the results of the five hypotheses (path coefficients) are different. When the *p*-value of the path coefficient is smaller than 0.05, it shows that the path coefficient is statistically significant and the hypothesis is supported.

Four out of the five hypotheses were supported. Specifically, attitude toward risk (β = 0.788, *p* < 0.001) and cognitive bias (β = 0.194, *p* = 0.033) have a significant positive influence on construction worker risk-taking behavior. Moreover, risk perception (β = −0.387, *p* < 0.01) and work condition (β = −0.281, *p* < 0.001) have a significant negative influence on construction worker risk-taking behavior. Lastly, safety climate (β = −0.104, *p* = 0.425) has no significant effect on risk-taking behavior.

### 4.3. Analysis on the Effect of Demographic Factors on Risk-Taking Behavior of Construction Workers

To examine whether risk-taking behavior of construction workers varied with demographics such as gender, age, education level, and marital status, the Kruskal-Wallis test was used. The results indicated that risk-taking behavior of construction workers varied with education level (χ^2^ = 8.302, df = 3, *p* < 0.05). However, no significant effect of gender, age, and marital status on the risk-taking behavior of construction workers was found.

## 5. Discussion

The proposed research model of construction worker risk-taking behavior was based on the relevant literature and tested using a questionnaire survey with structural equation modeling. The findings of this study provide empirical evidence that work conditions, attitude toward risk, cognitive bias, and risk perception have a significant influence on construction worker risk-taking behavior, whereas safety climate does not have a significant influence. This study contributes to the relevant literature by considering safety climate, work conditions, attitude toward risk, cognitive bias, and risk perception to explain construction worker risk-taking behavior. 

### 5.1. Attitude toward Risk

In this study, among tested constructs, risk-taking behavior was mostly affected by attitude toward risk (c.f., Figure 2); specifically, attitude toward risk was found to have a positive influence on risk-taking behavior. Moreover, the highest correlation was found between attitude toward risk and risk-taking behavior (c.f., Table 4). These findings are consistent with the qualitative study of Low et al. [24] who reported that super-safe workers who did not have incident records for the past five years held a negative attitude toward risk. According to the expected utility theory, which is about how people make optimal decisions under risk [55], a risk-seeker who holds a positive attitude toward risk tends to take risks, whereas people who have a negative attitude toward risk are less likely to take risks. This study provides supporting evidence for this theory in the context of construction safety. However, no studies have considered attitude toward risk in explaining construction worker risk-taking behavior. Thus, the present study filled this research gap and applied theoretical knowledge to the relevant research areas. The importance of attitude toward risk in construction worker risk-taking behavior was accentuated. In practice, safety management can make use of this finding in developing effective safety measures to reduce construction worker risk-taking behavior. For example, safety supervisors can provide close safety supervision to construction workers who have a high level of attitude toward risk to avoid their risk-taking behavior, because such workers tend to take risks at work. 

### 5.2. Cognitive Bias

The result of this study showed that the cognitive bias of construction workers positively influences their risk-taking behavior. Thus, the higher the level of cognitive bias, the more they perform risk-taking behavior. Previously, Johnson et al. [56] found that people consider cognitive bias an irrational confidence to cope with daily risks, including those at work, and that this bias can counterintuitively improve decision making. Moen and Rundmo [57] conducted a study in Norway to examine the predictors of unrealistic optimism among risk takers, such as firefighters. They found that safety attitude, control, and anxiety can be used to predict unrealistic optimism. However, no studies have been conducted to identify the predictors of cognitive bias among construction workers. The present study found that construction workers who have a high level of cognitive bias tend to take risks at work. In the future, more research efforts should be made to understand the cognitive bias of construction workers. 

### 5.3. Risk Perception

Risk perception is an important factor affecting the behaviors of people in different aspects. For example, in transportation safety, Harbeck and Glendon [58] discovered that young drivers who have a high level of risk perception tend not to engage in risky driving. In public health and safety, Nie et al. [59] indicated that the risk perception of patients can be used to predict health-promoting self-care behaviors, such as diet, exercise, self-monitoring of blood glucose, adherence to treatment, foot care, interpersonal relationships, and personal health responsibility. In construction safety, qualitative studies have reported that the risk perception of construction workers is one of the causes for their risk-taking behavior [13,24]. For instance, construction workers do not take risks, such as not wearing safety shoes, when they believe that not doing so can result in severe injuries. The present study provides quantitative evidence for the findings of Low et al. [24]. Specifically, the risk perception of construction workers has a negative influence on their risk-taking behavior. Rundmo [60] applied the suggestions of Sjöberg [61] that risk perception can be classified into two components, namely, cognitive and affective, to understand the occupational risk behavior of workers in the company Norsk Hydro. Although risk perception was not classified into two components in this study, the influence of risk perception on construction worker risk-taking behavior and its importance for construction safety were highlighted. In future studies, the effects of the components of risk perception on construction worker risk-taking behavior should be examined. The finding that risk perception has a negative influence on construction worker risk-taking behavior can be beneficial to the construction industry. Practically, safety management can provide more safety training and media related to work risks, such as falling and slipping from heights and tripping or falling on the same level, to construction workers who have a low level of risk perception [62,63]. This method aims to enhance their risk perception and, in turn, reduce their risk-taking behavior [62,63].

### 5.4. Safety Climate

The hypothesis that safety climate has a negative influence on construction worker risk-taking behavior was not supported in this study. Despite our full and clear promise of complete anonymity and confidentiality, it is possible that the surveyed workers somewhat feared to accurately describe the safety climate of their work, and this may have contributed to hiding the potential relationship. This is possible because safety climate items were related to potentially sensitive aspects (c.f., Table 1). Furthermore, another possible reason for such a finding is that some factors serve as mediators in the relationship between safety climate and risk-taking behavior. Identifying these mediators is an interesting research topic that is worth receiving more research attention. Although the hypothesis was not supported, this study found that construction worker risk-taking behavior is negatively correlated with safety climate (c.f., Table 4). This finding suggests that construction companies should develop effective interventions to improve safety climate, such as organizing construction safety week carnivals and safety video competitions. With good safety climate, construction worker risk-taking behavior can potentially be reduced.

### 5.5. Work Conditions

Previous qualitative studies have supported the fact that poor work conditions, such as limited space, insufficient lighting, safety equipment unavailability, and debris problems, can make construction workers take risks at work [13,24]. This study examined the extent to which work conditions influenced construction worker risk-taking behavior. The findings show that work condition has a negative effect on construction worker risk-taking behavior. In other words, when good work conditions, such as sufficient lighting and space, can be provided to construction workers, they are less likely to take risks. This result emphasizes the importance of workplace management in encouraging construction safety. This finding is in line with that of Wu et al. [64], who found that an effective safety system for reducing the occurrence of construction accidents should include safety planning of environment and equipment. In practice, construction practitioners should pay attention to the provision of good work conditions for construction workers to work safely. Concerted authorities can offer financial support, such as small and medium enterprise (SME) sponsorship schemes, to construction companies [65]. In the SME sponsorship schemes, small- and medium-sized construction companies can be given a grant for purchasing work equipment, such as light-duty working platforms, to ensure that construction workers work with an appropriate working platform to avoid accidents. 

## 6. Conclusions

This study is the first to provide insights into construction worker risk-taking behavior with consideration of attitude toward risk, cognitive bias, risk perception, work conditions, and safety climate. Specifically, results showed that attitude toward risk and cognitive bias has a positive influence on construction worker risk-taking behavior, whereas risk perception and work conditions had a negative influence. Risk-taking behavior of construction workers was mostly affected by attitude toward risk among these factors. Although safety climate did not significantly influence construction worker risk-taking behavior, it was found to be negatively correlated with construction worker risk-taking behavior. Moreover, given the findings of this study, practical recommendations for reducing construction worker risk-taking behavior were discussed earlier. Despite the useful findings of this study for academia and practical applications, its limitations should be recognized. As the proposed research model in this study was only tested with Hong Kong construction workers, the findings of this study may not be generalized to other regions due to cultural differences. This study should be replicated with other populations to verify and generalize the research findings in the future.

## Figures and Tables

**Table 1:** Item contents of the constructs in the proposed model.

Constructs	Items	Item contents	References
Attitude toward risk (ATR)	ATR1	You like taking risks at work.	[29,30]
	ATR2	You think taking risks at work is a good idea.	
Cognitive bias (CB)	CB1	In a situation where labor and material resources are already mobilized, it is impossible to stop working for any safety considerations even if you know about the potential risks.	[31]
	CB2	You believe that you can prevent any kind of accident related to your work.	
	CB3	Your sufficient, relevant experience ensures that you will not be injured in the construction site.	
Risk perception (RP)		You think it is very risky if …	[18,32,33]
	RP1	you continue doing a typical task when you are feeling tired or fatigued.	
	RP2	you walk across wet ground where electrical wires or cables are laid out.	
	RP3	you do not wear a safety harness when working on a 1.5 m (5 ft) high scaffolding platform without a guardrail.	
Safety climate (SC)	SC1	You think that your supervisor or company provides adequate and rigorous safety supervision and support in your workplace.	[34,35]
	SC2	Your company has a firm commitment to safety by monitoring safety in the workplace.	
	SC3	Members of your team are very concerned about their own safety performance.	
	SC4	You feel great satisfaction whenever you have high safety performance.	
Work condition (WC)	WC1	You always secure the hook of your safety harness while working at heights because anchor points are available for hooking.	[29,36,37,38]
	WC2	You always work under sufficient lighting.	
	WC3	Given the restrictions in workplaces regarding the use of approved access ladders or working platforms, you always use proper ladders to carry out your work above ground.	
	WC4	During handling construction debris, safety gloves are always available for you.	
	WC5	Many safety equipment/devices required at work are always available on the spot or easy to obtain.	
Risk-taking behavior (RTB)		During the past 12 months,	[39,40]
	RTB1	you have always worked or walked across a guardrail-free working platform at a height of 2 m or more above ground without wearing a safety harness.	
	RTB2	you have always worked without using the required PPE, tools, or other working equipment.	
	RTB3	you have always worked at a height of 2 m or more above ground without anchoring your safety harness properly.	
	RTB4	you have always used any unapproved access ladders when working at a height of 1.5 m or more above ground.	
	RTB5	you have always refused to wear safety goggles and earplugs during cutting and hammering?	

**Table 2:** Summary of participant characteristics (*N* = 188).

Demographic Information	Number of Participants	Percentage (%)
Gender		
Male	156	83.0
Female	32	17.0
Age		
30 or Below	36	19.2
31–40	43	22.9
41–50	53	28.2
51–60	46	24.5
61 or Above	10	5.2
Education Level		
Primary School or Below	28	14.9
Lower Secondary	76	40.4
Higher Secondary	48	25.5
Diploma/Certificate	27	14.4
Degree or Above	9	4.8
Marital Status		
Single	43	22.9
Married	132	70.2
Divorced/Separated	11	5.9
Widowed	2	1.1

**Table 3:** Construct reliability and convergent validity.

Constructs	Items	FL	CR	AVE	Cronbach’s α
Attitude toward risk (ATR)	ATR1	0.784	0.852	0.744	0.836
	ATR2	0.934			
Cognitive bias (CB)	CB1	0.662	0.825	0.614	0.818
	CB2	0.861			
	CB3	0.813			
Risk perception (RP)	RP1	0.773	0.797	0.569	0.788
	RP2	0.662			
	RP3	0.820			
Safety climate (SC)	SC1	0.822	0.808	0.515	0.809
	SC2	0.636			
	SC3	0.651			
	SC4	0.746			
Work condition (WC)	WC1	0.770	0.843	0.521	0.840
	WC2	0.743			
	WC3	0.796			
	WC4	0.584			
	WC5	0.698			
Risk-taking behavior (RTB)	RTB1	0.801	0.858	0.549	0.868
	RTB2	0.710			
	RTB3	0.749			
	RTB4	0.741			
	RTB5	0.698			

**Table 4:** Inter-correlations among the constructs.

	ATR	CB	RP	SC	WC	RTB
**ATR**	0.86					
**CB**	0.18 *	0.78				
**RP**	−0.34 *	−0.27 *	0.75			
**SC**	−0.32 *	0.04	0.30 *	0.72		
**WC**	−0.29 *	−0.12 *	0.49 *	0.39 *	0.72	
**RTB**	0.51 *	0.10 *	−0.47 *	−0.19 *	−0.46 *	0.74

Note: Diagonal values are the square root of the AVEs; *: *p* < 0.05. The acronyms used are ATR (attitude toward risk), CB (cognitive bias), RP (risk perception), SC (safety climate), WC (work condition), and RTB (risk-taking behavior).
